# Is it feasible to measure intra-abdominal pressure using a balloon-tipped rectal catheter? Results of a validation study

**DOI:** 10.1007/s10877-022-00890-6

**Published:** 2022-07-30

**Authors:** Anneleen S. Staelens, Ann Heymans, Sigrid Christiaens, Niels Van Regenmortel, Wilfried Gyselaers, Manu L. N. G. Malbrain

**Affiliations:** 1 Department Obstetrics and Gynaecology, Ziekenhuis Oost Limburg, Genk, Belgium; 2 Department Medicine and Life Sciences, Hasselt University, Hasselt, Belgium; 3 Department of Intensive Care, Ziekenhuis Netwerk Antwerpen, ZNA Stuivenberg, Antwerp, Belgium; 4 Department of Physiology, Hasselt University, Hasselt, Belgium; 5 First Department of Anaesthesiology and Intensive Therapy, Medical University of Lublin, Jaczewskiego Street 8, 20-954 Lublin, Poland; 6 Medical Data Management, Medaman, 2440 Geel, Belgium; 7 International Fluid Academy, Dreef 3, 3360 Lovenjoel, Belgium

**Keywords:** Intra-abdominal pressure, Rectal measurement, Intra-vesical measurement, Validation, Abdominal hypertension, Monitoring, Validation

## Abstract

The gold standard to measure intra-abdominal pressure (IAP) is intra-vesical measurement via the urinary bladder. However, this technique is restricted in ambulatory settings because of the risk of iatrogenic urinary tract infections. Rectal IAP measurements (IAP_rect_) may overcome these limitations, but requires validation. This validation study compares the IAP_rect_ technique against gold standard intra-vesical IAP measurements (IAP_ves_). IAP_rect_ using an air-filled balloon catheter and IAP_ves_ using Foley Manometer Low Volume were measured simultaneously in sedated and ventilated patients. Measurements were performed twice in different positions (supine and HOB 45° elevated head of bed) and with an external abdominal pressure belt. Sixteen patients were included. Seven were not eligible for analysis due to unreliable IAP_rect_ values. IAP_rect_ was significantly higher than IAP_ves_ for all body positions (p < 0.01) and the correlation between IAP_ves_ and IAP_rect_ was poor and not significant in each position (p ≥ 0.25, R^2^ < 0.6, Lin’s CCC < 0.8, bias − 8.1 mmHg and precision of 5.6 mmHg with large limits of agreement between − 19 to 2.9 mmHg, high percentage error 67.3%, and low concordance 86.2%). Repeatability of IAP_rect_ was not reliable (R = 0.539, p = 0.315). For both techniques, measurements with the external abdominal pressure belt were significantly higher compared to those without (p < 0.03). IAP_rect_ has important shortcomings making IAP estimation using a rectal catheter unfeasible because the numbers cannot be trusted nor validated.

**Supplementary Information:**

The online version contains supplementary material available at 10.1007/s10877-022-00890-6.

## Introduction

Intra-abdominal pressure (IAP) is the steady state pressure inside the abdominal cavity and is normally less than 7 mmHg in healthy adults with higher physiological baseline levels (9 to 14 mmHg) in morbidly obese patients [1, 2]. Critically ill patients usually have baseline IAP of approximately 10 mmHg [3]. Intra-abdominal hypertension (IAH) is defined by the  Abdominal Compartment Society (WSACS), formerly known as the World Society of the Abdominal Compartment Syndrome (www.wsacs.org) [4, 5], as a sustained increased in IAP ≥ 12 mmHg, and abdominal compartment syndrome as IAP > 20 mmHg with new onset organ failure [6–8]. A pathologically increased IAP is often seen in critically ill patients and this may have detrimental consequences such as acute renal failure, hemodynamic instability, inadequate ventilation and decreased blood flow to organs [9, 10]. It has been suggested that gestational complications such as preeclampsia might also be associated with intra-abdominal hypertension [11–13], making IAP an important consideration in obstetric (patho) physiology as well.

Urinary bladder pressure measurement, by using a FoleyManometer Low Volume (FMLV), is recognised as the gold standard to measure IAP [14]. This technique is easily applicable in catheterized patients, but its use is restricted in ambulatory settings because of risks of iatrogenic urinary tract infections. The abdomen behaves according to Pascal’s Law, thus rectal pressure measurements proximal of the pelvic floor muscles should also represent IAP similar to intravesical pressure. Rectal pressures are used routinely as estimates for IAP during urodynamic studies to calculate transmural detrusor muscle pressure (intravesical pressure minus IAP measured rectally) [15–17]. From a theoretical point-of-view, measurement using a rectal catheter seems less invasive and could potentially be used in ambulatory settings and in pregnant patients, however, validation of this technique is required. This validation study compares the rectal intra-abdominal pressure (IAP_rect_) technique against standard intra-vesical IAP measurements (IAP_ves_).

## Material and methods

### Ethical approval

The study was conducted at the Ziekenhuis Netwerk Antwerpen (ZNA Campus Stuivenberg, Antwerp, Belgium) in accordance with the study protocol, the Declaration of Helsinki and applicable regulatory requirements. The study was approved by the local Institutional Review Board and Ethics Committee of ZNA (Antwerp, Belgium) (EC Approval number 3001) and Ziekenhuis Oost-Limburg (Genk, Belgium) (EC 12/084U). Oral and written informed consent was obtained from the relatives of all patients and there were no deviations from standard clinical practice.

### Patient selection

Sedated and ventilated patients admitted to the ICU (Ziekenhuis Netwerk Antwerpen, ZNA Campus Stuivenberg, Antwerp, Belgium) were included from December 2014 to May 2015. Exclusion criteria were patients younger than 18 years and those in whom there was a medical contraindication for rectal or urinary bladder catheterisation. Demographic data were recorded for all patients.

### Pressure measurements

In most patients, a urinary catheter was already in place with a Foley Manometer Low Volume (FMLV, Holtech, Medical, Charlottenlund, Denmark) attached. If not, a urinary catheter was inserted prior to FMLV attachment. In case of an empty urinary bladder or the presence of air-bubbles obstructing a continuous fluid column in the FMLV, 20 ml of 0.9% sterile sodium chloride solution was injected via the FMLV urine sample port using an aseptic technique. Baseline IAP was measured in the supine position using the FMLV (IAP_ves_) with the zero-reference point in the midaxillary line at the level of the iliac crest (as recommended by WSACS) [14, 18]. IAP was noted at end-expiration, when the meniscus of the fluid column had stabilized and oscillated with the breathing efforts.

The IAP_rect_ was measured using a rectal T-DOC 7Fr air-filled balloon catheter (Laborie Medical Technologies, Mississauga, Canada) connected to a computer displaying the IAP (Audact Pro database version 7.11, Ellipse Andromeda, Urotex, The Netherlands) (ESM Fig. 1). The balloon was inflated with air using a switch, zeroed at atmospheric pressure and inserted 15 cm into the rectum after digital rectal palpation to remove impacted faeces. The catheter was attached to the patient’s leg to prevent displacement.

### Study protocol

**Fig. 1:**
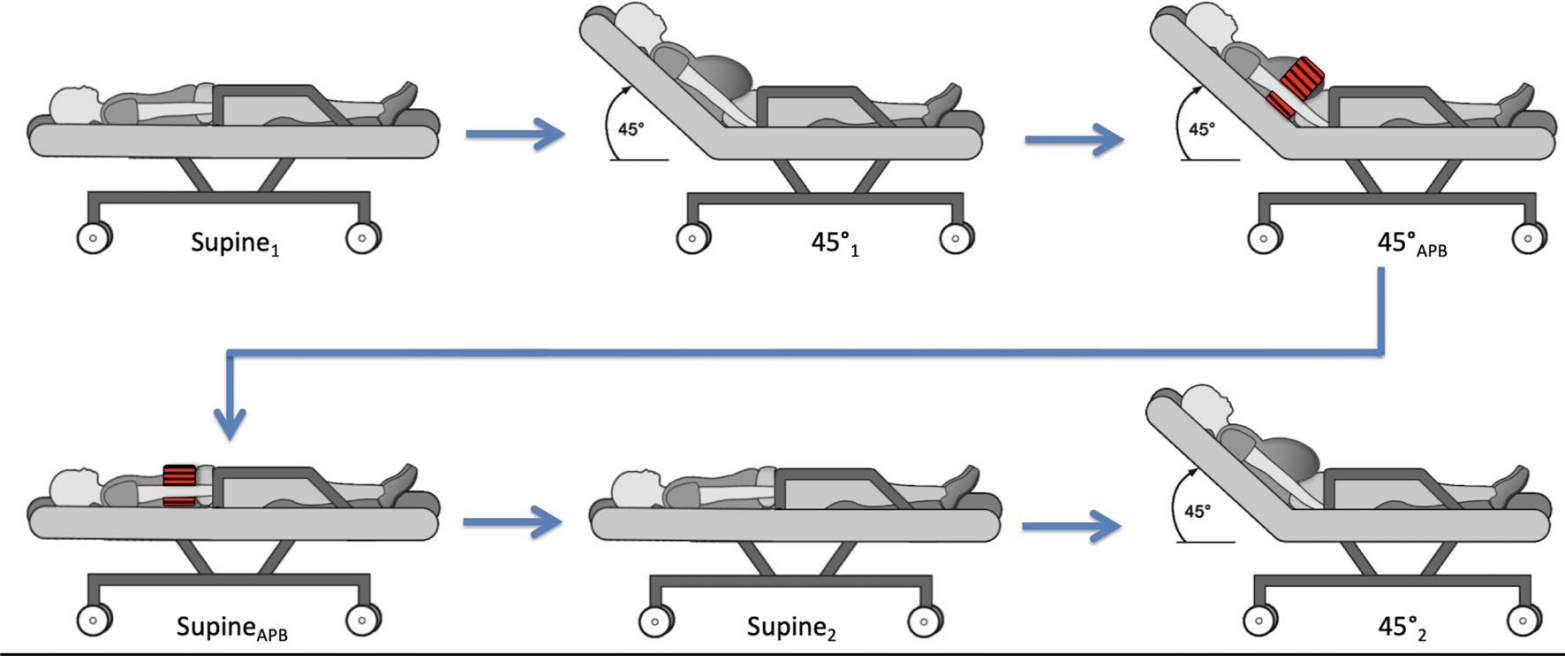
Standardized protocol to measure intra-abdominal pressure. Intra-abdominal pressure measurements were performed in different positions: supine position (Supine_1_ and Supine_2_) and 45° semirecumbent (45°_1_ and 45°_2_) without an external abdominal pressure belt, and 45° semirecumbent (45°_APB_) and supine position (Supine_APB_) with an external abdominal pressure belt (marked with a red spot)

IAP is most accurate when measured in a supine position [8, 16]. To validate the accuracy of IAP_rect_ with increasing IAP, measurements were performed in 2 positions in an attempt to artificially increase IAP; the 45° elevated head of bed semirecumbent position, followed with an external abdominal pressure belt (similar to that used by surgeons to prevent incisional hernias). The abdominal belt was put on manually and fastened with a velcro tape and was not released during the protocol. IAP_ves_ and IAP_rect_ were measured simultaneously according to a standardized protocol (Fig. 1). All positions (except the application of the external abdominal pressure belt) were repeated twice, including the insertion of the rectal catheter.

### Statistical analysis

All statistical analyses were performed using SPSS 28.0 software (SPSS inc., Chicago, USA). Results of continuous data that were normally distributed are presented as mean (± SD) unless otherwise stated. Mean values were compared using a paired or independent sample Student’s *t* test whenever appropriate. A p value of < 0.05 was considered significant. Paired measurements by the two different IAP methods were compared using five different statistical methods.

First, correlations between IAP_ves_ and IAP_rect_ were evaluated using univariate linear regression analysis and Pearson correlation coefficient. Two methods are considered equal if the line of identity crosses the origin of X and Y-axis and if R^2^ (R = Pearson’s correlation coefficient) is > 0.6. Second, we calculated bias (mean difference between reference technique IAP_ves_ and IAP_rect_), precision (SD of the bias) and limits of agreement (bias ± 1.96 × precision) according to Bland and Altman. We followed the Abdominal Compartment Society (WSACS, www.wsacs.org) guidelines and recommendations for research from the international conference of experts on intra-abdominal hypertension and abdominal compartment syndrome on validation of new IAP technology against the gold standard [19]. The bias should be maximal 1 mmHg with a precision less than 2 mmHg to allow two techniques to be used interchangeably. Using a t test and assuming equal standard deviations and an anticipated mean for IAP_ves_ around 13.6 ± 3.1 mmHg in the supine position and assuming rectal pressures overestimating IAP_ves_ with a mean IAP_rect_ of 18 to 19 mmHg, and assuming a type I error rate alpha of 0.05, with a type II error rate or power (1-β) of 80% an adequate sample size should be 10 to 16, depending on a mean IAP_rect_ of 19 vs. 18 mmHg respectively. Power and sample size calculation was performed with Clincalc (https://clincalc.com/stats/samplesize.aspx).

Third, the percentage error (two times precision of the bias divided by the mean of the reference IAP technique) was calculated as described previously [19]. Based on previous reports, the percentage error for IAP should be less than 35%.

Fourth, Lin’s Concordance Correlation Coefficient (CCC) was calculated as previously described as an extra method for comparing two measurements (rectal vs. vesical) of the same variable (IAP). Ideally the CCC should be above 0.94.

Fifth, the ability of IAP_rect_ to track changes or trends in IAP_ves_ was assessed by plotting ΔIAP_rect_ against ΔIAP_ves_ during the same time interval (four quadrants trend plot). The concordance is calculated as the percentage of pairs with the same direction of change after exclusion of pairs with both a ΔIAP_rect_ and ΔIAP_ves_ ≤ 2.5 mmHg (or less than 15% of change) or with either ΔIAP_rect_ and ΔIAP_ves_ equal to zero. Based on clinical relevance, the concordance should be > 90% after exclusion of the pairs falling within the exclusion zone with ΔIAP from − 2.5 to + 2.5 mmHg.

## Results

### Patient demographics

**Fig. 2:**
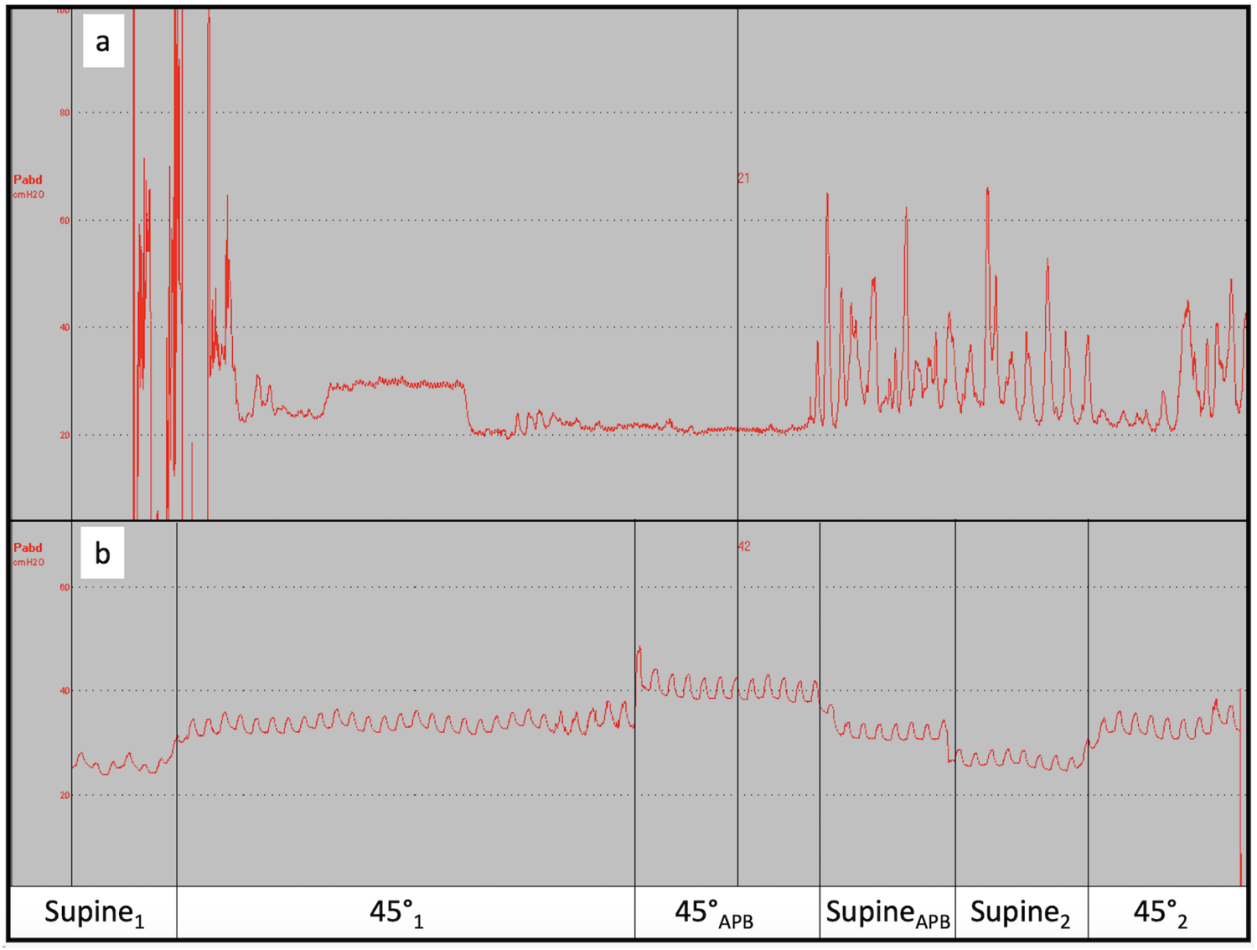
Output of a rectal intra-abdominal pressure measurement. Intra-abdominal pressure measurement in cmH_2_O (y-axis) presented over time (x-axis). **a** invalid measurement due to improper placement of the catheter (first part) and active abdominal muscle contraction (pushing) (last part). **b** valid measurement in which the influence of the breathing is observed: supine position (Supine_1_ and supine_2_) and 45° semirecumbent (45°_1_ and 45°_2_) without an external abdominal pressure belt, 45° semirecumbent (45°_APB_) and supine position (Supine_APB_) with an external abdominal pressure belt

Sixteen patients were included, of whom 7 (43.8%) were not eligible for analysis as all IAP_rect_ measurements were unreliable due to IAP_rect_ values out of physiological range (> 40 mmHg) or strongly fluctuating (> 50%). These patients were found to have profound diarrhoea (n = 1), faecal impaction (n = 2), abdominal muscle contractions in a subconscious patient (n = 1) and difficult placement of the catheter due to anal skin tags and haemorrhoids (n = 1) or morbid obesity (n = 2). An example of both invalid and valid IAP_rect_ measurement is shown in Fig. 2. The men/women ratio of the of the 9 remaining patients (56.2%) eligible for further analysis was 7/2, with mean age 59.0 ± 13.5 years and mean BMI 26.9 ± 6.8 kg/m^2^. Patients were admitted to ICU for sepsis (n = 3), vascular disease, liver cirrhosis, exacerbation of COPD, pneumonia (n = 4), and major trauma (n = 2).

### Effect of interventions (position and pressure belt)

**Fig. 3:**
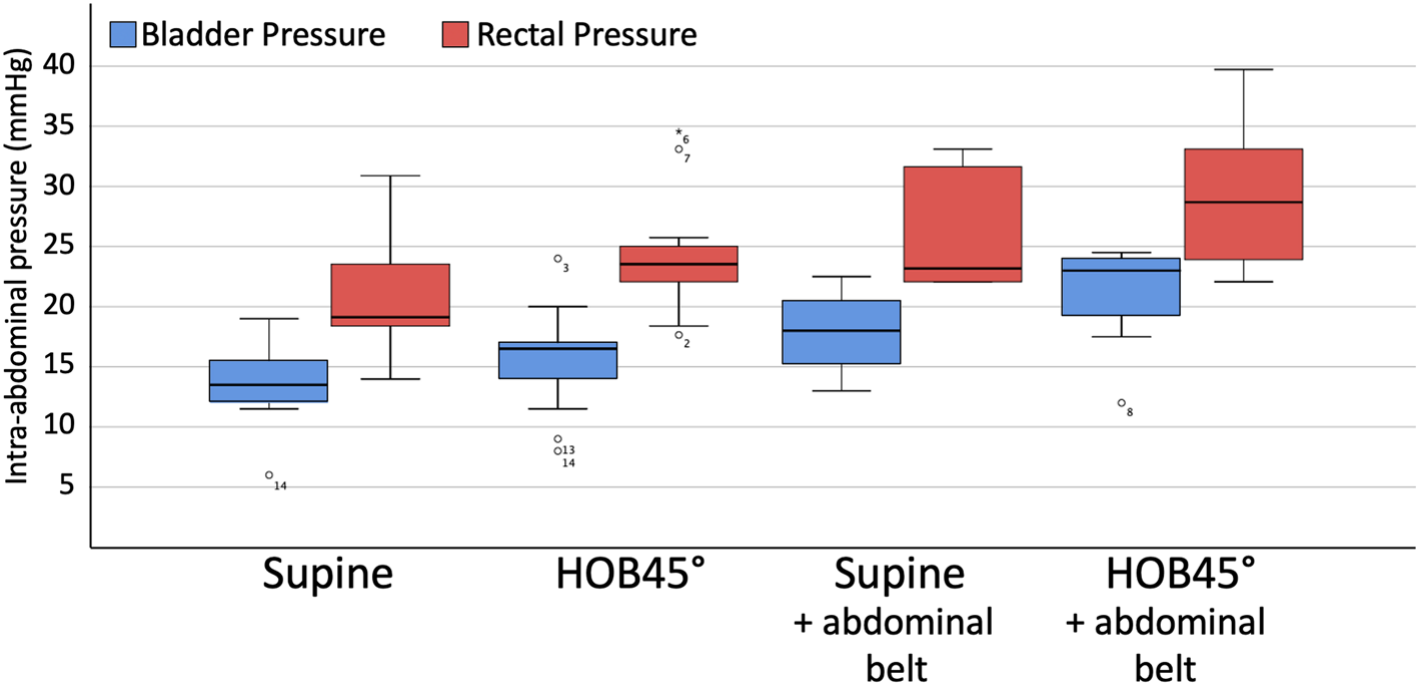
Boxplots comparing vesical (IAP_ves_) and rectal intra-abdominal pressure (IAP_rect_) measurements in different body positions. Box and whisker plots comparing IAP_ves_ and IAP_rect_ in different body positions. The error bars are the 95% confidence interval, the bottom and top of the box are the 25th and 75th percentiles, the line inside the box is the 50th percentile (median), and any outliers are shown as open circles, p value < 0.05 for all vesical vs. rectal IAP comparisons

Four patients had only one set of measurements and the external abdominal pressure belt was contra-indicated in one patient because of rib fractures, therefore there were in total 14 paired measurements without and 8 measurements with the abdominal belt in supine position. One patient was not able to be put in HOB 45° position because of pain, therefore there were in total 13 paired measurements without and 7 measurements with the abdominal belt in HOB 45° position. Table 1 shows the mean IAP_ves_ and IAP_rect_ in both positions, and with or without the external abdominal pressure belt. IAP_ves_ was lowest in the supine position (13.6 ± 3.1 mmHg), however, not significantly different from measurement obtained in the semi-recumbent HOB 45° position (15.7 ± 4.4 mmHg, p = 0.103). Measurements with the abdominal pressure belt were significantly higher compared to those without (p < 0.03). Figure 3 shows a boxplot of the median IAP_ves_ and IAP_rect_ values in the different body positions and with or without the external abdominal pressure belt.

**Table 1:** Correlation between intravesical (IAP_ves_) and intrarectal (IAP_rect_) pressure in different body positions and with and without external abdominal pressure belt (APB)

	Supine (n = 14)	HOB45° (n = 13)	Supine_APB_ (n = 8)	HOB45°_APB_ (n = 7)	Total (n = 42)
IAP_ves_ (mmHg)	13.6 ± 3.1	15.7 ± 4.4	17.9 ± 3.4	20.9 ± 4.6	16.3 ± 4.5
IAP_rect_ (mmHg)	20.8 ± 5.0	24.4 ± 4.8	26.1 ± 5.1	29.2 ± 6.8	24.3 ± 5.9
PCC	0.159	0.344	0.283	0.152	0.451
R^2^	0.025	0.119	0.080	0.023	0.204
Lin’s CCC	0.055	0.118	0.085	0.064	0.198
p value	0.588	0.250	0.497	0.745	0.003

Data are presented as mean ± standard deviation. Correlations reported between IAP_ves_ and IAP_rect_

*APB* abdominal pressure belt, *CCC* Lin’s concordance correlation coefficient, *HOB45*° 45° head-of-bed (HOB) semirecumbent position, *IAP*_*ves*_ intravesical pressure, *IAP*_*rect*_ intrarectal pressure, *PCC* pearson correlation coefficient (R)

IAP_rect_ in the supine position was significantly lower compared to measurements in the semi-recumbent position (20.8 ± 5.0 mmHg versus 24.4 ± 4.8 mmHg (p = 0.002) for supine and semirecumbent position, respectively) and measurements with the abdominal pressure belt were significantly higher than without, (p = 0.032 and p = 0.003 for IAP_rect_ in supine and semi-recumbent positions, respectively).

### Correlation between bladder and rectal pressure measurement techniques

**Fig. 4:**
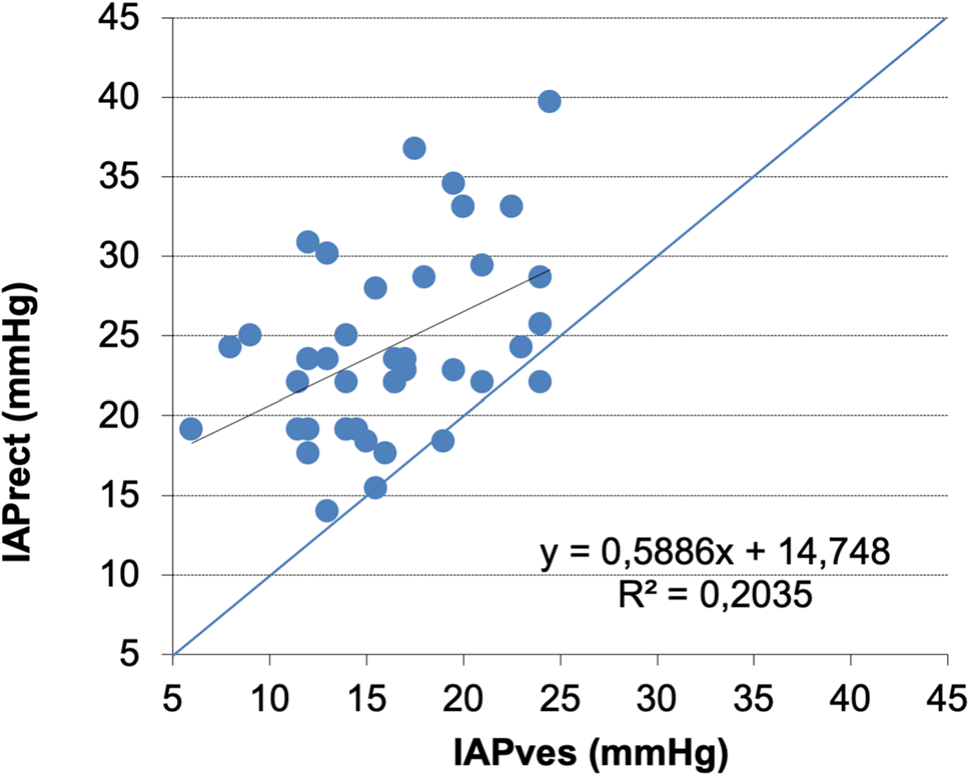
Correlation scatter plot comparing vesical and rectal intra-abdominal pressure measurements. Correlation plot for all IAP_ves_ and IAP_rect_ comparisons in different positions (supine, HOB45°) and with/without abdominal pressure belt (n = 42). Line of identity in blue and linear regression line in black. IAP_rect_: rectal intra-abdominal pressure; IAP_ves_: vesical intra-abdominal pressure

**Fig. 5:**
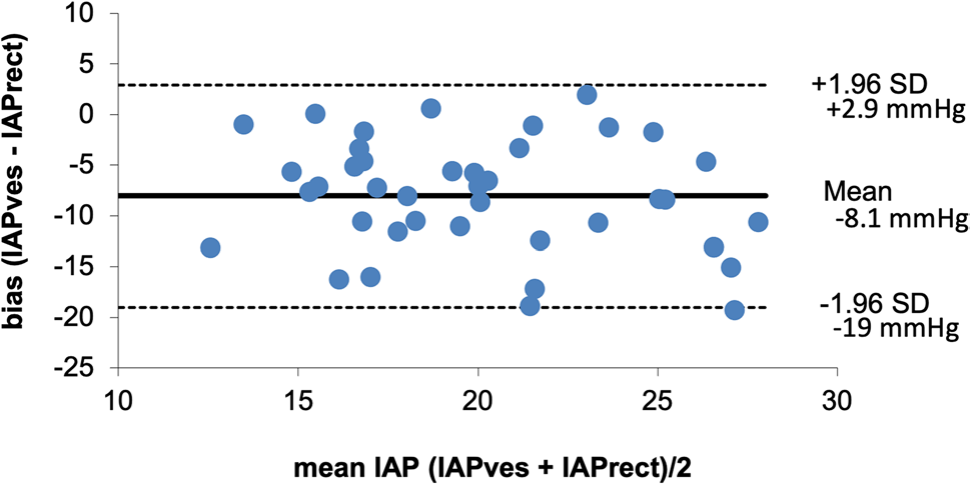
Bland and Altman plot comparing vesical and rectal intra-abdominal pressure measurements. Aggregated Bland and Altman plot for the mean difference between all IAP_rect_ and IAP_ves_, in different body positions (supine and HOB 45°) and with/without abdominal Pressure belt (n = 42), and their 95% limits of agreement. IAP_rect_: rectal intra-abdominal pressure; IAP_ves_: vesical intra-abdominal pressure

Correlation (Pearson and Lin concordance correlation coefficient) between IAP_ves_ and IAP_rect_ was poor (Table 1, Fig. 4, and ESM Fig. 2). An aggregated Bland and Altman analysis for IAP_rect_ versus IAP_ves_ (n = 42) shows an abnormal bias of − 8.1 mmHg and precision of 5.6 mmHg with large limits of agreement between − 19 and 2.9 mmHg (Fig. 5, and ESM Fig. 3). The percentage error (LA divided by mean IAP) was 67.3% and too high (should be below 35%) (Table 2).

**Table 2:** Aggregated Bland and Altman analysis comparing IAP_ves_ with IAP_rect_ measurements in different body positions (supine and HOB 45°) and with/without abdominal Pressure belt (n = 42)

IAPves (mmHg)	IAPrect (mmHg)	IAPmean (mmHg)	Mean bias (mmHg)	SD (precision)	LLA	ULA	PE (%)
16.3 ± 4.5	24.3 ± 5.9	20.3 ± 4.5	− 8.1	5.6	− 19.0	2.9	67.3

SE	SELA	L95%CI	U95%CI	L95%CILLA	U95%CILLA	L95%CIULA	U95%CIULA
0.9	1.5	− 9.8	− 6.4	− 22.0	− 16.1	0.0	5.9

Data expressed in mmHg

*Bias* IAPves−IAPrect, *IAP* intra-abdominal pressure, *IAPmean* mean IAP = (IAPves + IAPrect)/2, *IAPrect* intra-rectal pressure, *IAPves* intra-vesical pressure, *L95%CI* lower 95% confidence interval, *L95%CILLA* lower 95% confidence interval of the lower limit of agreement, *L95%CIULA* lower 95% confidence interval of the upper limit of agreement, *LLA* lower limit of agreement, *PE* percentage error (limits of agreement divided by mean IAP), *SD* standard deviation, *SE* standard error, *SELA* standard error limits of agreement, *U95%CI* upper 95% confidence interval, *U95%CILLA* upper 95% confidence interval of the lower limit of agreement, *U95%CIULA* upper 95% confidence interval of the upper limit of agreement, *ULA* upper limit of agreement

**Fig. 6:**
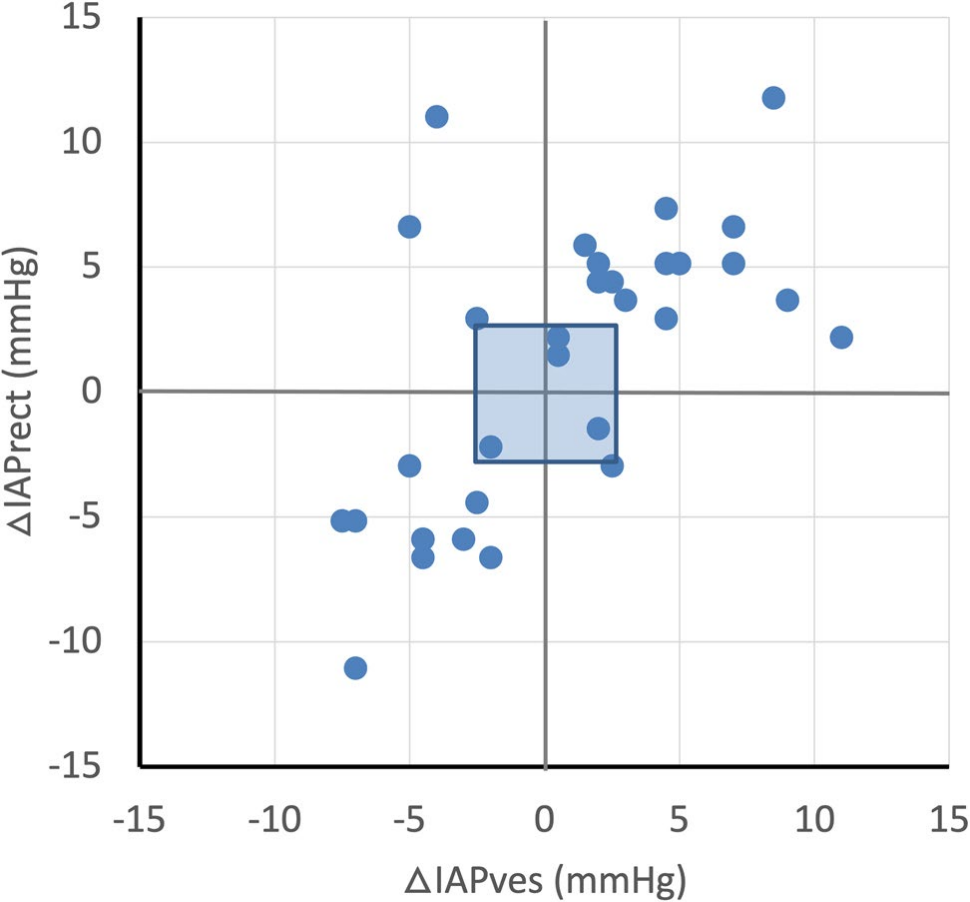
Four quadrant concordance plot looking at changes in IAP. Four quadrants trend plot for 33 paired measurements of ΔIAP_ves_ and ΔIAP_rect_. From the 33 initial paired measurements, 4 pairs were excluded because either ΔIAP was ≤  ± 2.5 mmHg or equal to zero (exclusion zone, blue shaded area). The calculated level of concordance was 86.2%. See text for explanation

IAP_rect_ was significantly higher than IAP_ves_ for all positions (p < 0.001) and reached the threshold of IAH (≥ 12 mmHg) in all patients, against 64.3% for the IAP_ves_ measurements. The IAP difference when moving from supine to 45° HOB semi-recumbent position was different between the two techniques: 1.2 ± 3.1 mmHg versus 3.5 ± 3.1 mmHg, for IAP_ves_ and IAP_rect_, respectively (p = 0.046). The concordance was insufficient (86.2%) after exclusion of the pairs falling within the exclusion zone with ΔIAP from -2.5 to + 2.5 mmHg (Fig. 6).

There was a no correlation between Supine_1_ and Supine_2_ (Fig. 1) for IAP_rect_ (R = 0.539, p = 0.315). When repeating the protocol, IAP_rect_ was out of physiological range (> 40 mmHg) or unstable in 4/7 patients that were not included for further analysis (57.1%).

## Discussion

### Correlation between bladder and rectal pressure measurement techniques

Various techniques have been developed to measure IAP, of which the intra-vesical approach is regarded as the gold standard, but it is relatively invasive in an ambulatory setting. There is a need for minimally- or non-invasive techniques of IAP monitoring, especially in obstetrics. Recently different techniques like microwave reflection and transient radar method have been suggested however these are not yet available for clinical use [20, 21]. IAP estimation via rectal measurements seems, at least from a theoretical or hypothetical point-of-view, appropriate and feasible in the pregnant population because of the low-infection risk and lower risk of trauma compared to vesical measurements. However, taking into account the many disadvantages and limitations we cannot recommend its routine ambulatory use, as will be discussed further.

This validation study found that IAP_rect_ is higher when compared to IAP_ves_, and thus may overestimate the true IAH incidence. Furthermore, the IAP differences and trend evolution after position change, or the application of an external abdominal pressure belt, are not similar to the gold standard technique. Correlation was poor, concordance was low, percentage error was too high, and Bland and Altman analysis showed too large LA, hence the two techniques cannot be used interchangeably. We also observed a very high failure rate in obtaining a reproducible IAP_rect_ measurement.

### Effect of body position

Change in body position has a significant impact on IAP measurement [22, 23]. We found that HOB elevation increased both the IAP_rect_ and IAP_ves_, (Fig. 3) which is in keeping with results from the literature. Previous studies confirmed that even a slight elevation in HOB results in a clinically apparent increase in IAP measured through the bladder [2, 22, 23]. Similar investigations were performed to check the impact of body position on IAP measurement not only through the bladder, but also through the stomach (intra-gastric pressure) [24]. HOB elevation increases the intra-gastric pressure (IGP) as well as the intra-bladder pressure, however, the IGP changes were observed to a smaller degree compared to IAP_ves_. In contrast to these studies that have investigated the impact of the upper body position on IAP measurement, the impact of the lower body position on IAP measurement has been assessed recently [25]. In this experiment, IAP measurement through the vagina and (in some cases) rectum at supine, low lithotomy, and high lithotomy positions were evaluated. Based on the results of this study, there is no clinically remarkable change in IAP when the legs are positioned differently. However, the IAP with the patient’s legs in the supine position were lower compared to the low and high lithotomy positions.

### Strengths and limitations of the study

This study is the first to attempt validation of IAP_rect_ measurements against the gold standard IAP_ves_ in an ICU-setting [9]. The inclusion of sedated patients, in whom confounding variables are lower, and the strict protocol are strengths of this study [26]. However, the small sample size, the poor description of patient demographics, high drop-out rate and the incompletely performed protocol due to patient or technique related issues are weaknesses and may have underpowered our study results.

The results from this validation study are similar to a study by McCarthy et al., who validated IAP_rect_ in 12 patients but found excessively high or unreliable values in 4 patients (33.3%) due to abdominal traction and technical difficulties on catheter insertion. They concluded that the rectal catheter should be inserted at least 10 cm deep to prevent pressure changes inside the rectum that may result in overestimated readings [27].

Significantly higher IAP_rect_ measurements were observed compared to IAP_ves_, even when IAP is within the physiological range, and as a result IAP_rect_ over-diagnoses IAH. This is in keeping with IAP_rect_ obtained with a fluid-filled rectal catheter balloon in which residual faecal mass can block the catheter-tip opening leading to overestimation of IAP [16]. Correcting this overestimation with a correction factor or the use of a different reference range might not be appropriate as there was no significant correlation between supine_1_ and supine_2_. Also, after re-insertion of the rectal catheter, measurement was not repeatable in more than half of the patients. This is in agreement with the results of Lacey et al., who evaluated different indirect techniques against invasive direct IAP measurement in rabbits [28]. Regression analysis showed good correlation with measurements performed in the inferior vena cava (R = 0.87) and the urinary bladder (R = 0.85), but not with intrarectal measurements (R = 0.10) [28]. On the contrary, Shafik et al. found IAP_rect_ to be similar to direct IAP measurement [15]. Note that IAP_rect_ was measured using a fluid filled rectal catheter.

### Limitations of rectal pressure measurement

Several factors may affect IAP_rect_ measured via an air-filled balloon. First, previous studies showed that body temperature is higher in the rectum compared to the urinary bladder [29, 30]. In the present study a small amount of air (at ambient temperature) was used to fill the rectal balloon and air is very sensitive to temperature changes. The higher rectal temperature will result in an increased air temperature in the balloon. In relation to the constant volume this may lead to a significantly increase in rectal pressure measured via the balloon-tipped catheter.

Second, the muscles in the rectum are stronger than the muscles in the urinary bladder. Physiologically, each rectal manipulation and filling stimulates the contraction of the rectal muscles. In a similar way, the insertion and filling of the (even small) balloon could trigger this reflex. Additionally, IAP_rect_ is also affected by the internal anal sphincter tension which contributes about 85% of the pressure in the anal canal [31]. Studies in healthy volunteers showed a significant increase in the internal anal sphincter tension followed by an increase in IAP_rect_ after insertion of an artificial manometer for IAP_rect_ measurement [31, 32]. Therefore, we can assume that rectal insertion of the T-DOC 7Fr air-filled balloon catheter can increase IAP_rect_ per se.

Third, the high failure rate experienced was largely due to IAP_rect_ measuring values out of the physiological range, or due to difficulties with rectal catheter insertion. These excessive IAP values may be caused by interference from faecal masses or bowel movements on the catheter-tip opening, or an incorrect catheter position at the level of the rectal sphincter. Measuring pressure at the level of the rectal sphincter is used in anorectal manometry but it does not yield information regarding true IAP.

Fourth, as pregnant women have an increased risk of constipation and haemorrhoids, therefore, this IAP measurement technique is not suitable to perform in a pregnant population. Laxatives might help to overcome the problem of obstructing stool; however, this is not appropriate in an ambulatory setting.

Fifth, although we perceive rectal pressure measurement as being less or even minimal invasive compared to bladder pressure measurement because of the virtual absence of infection risk it must be noted that rectal manipulation can induce parasympathetic hyperactivity with severe bradycardia and cardiac arrhythmias [33].

Sixth, continuous IAP (CIAP) monitoring is the future [34, 35] and even if rectal pressures would be accurate and comparable to bladder pressures (which was not the case) continuous IAP_rect_ monitoring would be difficult because of probe positioning, displacement, and faecal interference.

## Conclusion

This validation study found that IAP_rect_ is higher when compared to IAP_ves_. The observed IAP changes induced by position change or the application of an external abdominal pressure belt are not similar when measured via the rectum versus the bladder. Overall correlation was poor, concordance was low, percentage error was too high, and Bland and Altman analysis showed too large LA, hence the two techniques cannot be used interchangeably. IAP_rect_ has important shortcomings making IAP estimation using a rectal catheter unfeasible, largely because the numbers cannot be trusted nor validated. Future studies should be done in different patient populations and/or ambulatory patients to confirm or refute our findings.

## Supplementary Information

Below is the link to the electronic supplementary material.

Supplementary file1 (DOCX 4515 kb)

## Data Availability

The datasets used and/or analyzed during the current study are available from the first author on reasonable request.

## Abbreviations

ACS: Abdominal compartment syndrome
IAP: Intra-abdominal pressure
IAH: Intra-abdominal hypertension
IAP_ves_: Vesical intra-abdominal pressure
IAP_rect_: Rectal intra-abdominal pressure
HOB: Head of bed
FMLV: Foley Manometer Low Volume
LA: Limits of agreement
COPD: Chronic obstructive pulmonary disease
ICU: Intensive care unit
