# Supplementary material for: Is it feasible to measure intra-abdominal pressure using a balloon-tipped rectal catheter? Results of a validation study
Source: J Clin Monit Comput. 2022 Jul 30;37(1):287–96. doi: 10.1007/s10877-022-00890-6 (PMC9852189; doi:10.1007/s10877-022-00890-6)
Supplement: Supplementary file 1 — Supplementary file1 (DOCX 4515 kb) [file 10877_2022_890_MOESM1_ESM.docx]

**Electronic supplemental material on "Is it feasible to measure intra-abdominal pressure using a balloon-tipped rectal catheter? – Results of a validation study"**

Journal Clinical Monitoring and Computing

Submission ID 0d66a33e-e767-400a-921b-e61070c02772

**ESM Figure 1.** Air-charged 7 Fr T-DOC rectal balloon-tipped catheter (Laborie Medical Technologies, Mississauga, Canada) connected to a computer displaying the IAP (Audact Pro database version 7.11, Ellipse Andromeda, Urotex, Rhenen, The Netherlands).

**ESM Figure 2.** Correlation plots for IAP_ves_ and IAP_rect_ comparisons in different positions (supine, HOB45°) and with/without abdominal Pressure belt (n=42).

Panel A. Correlation plot for IAP_ves_ and IAP_rect_ comparisons in supine position (n=14)


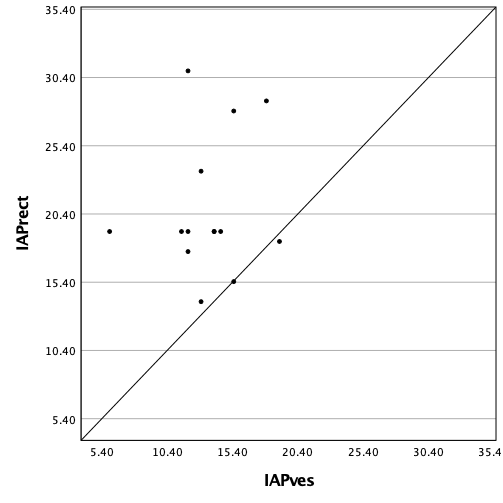


Panel B. Correlation plot for IAP_ves_ and IAP_rect_ comparisons in head of bed (HOB) 45° position (n=13)


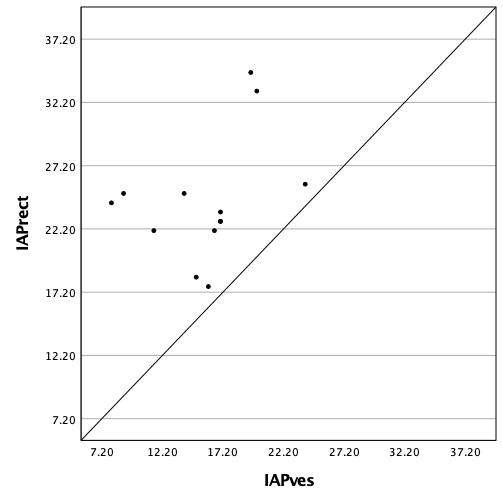


Panel C. Correlation plot for IAP_ves_ and IAP_rect_ comparisons in supine position with abdominal Pressure belt (n=8)


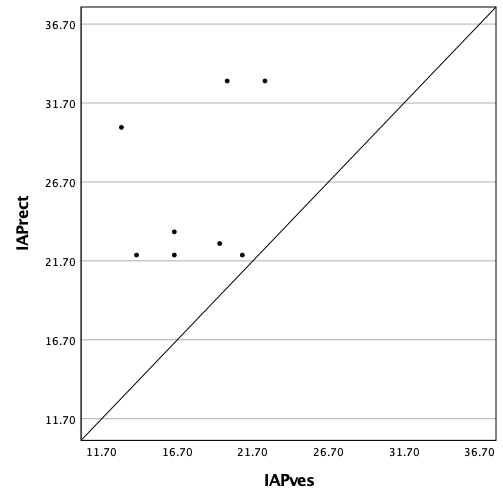


Panel D. Correlation plot for IAP_ves_ and IAP_rect_ comparisons in head of bed (HOB) 45° position with abdominal Pressure belt (n=7)


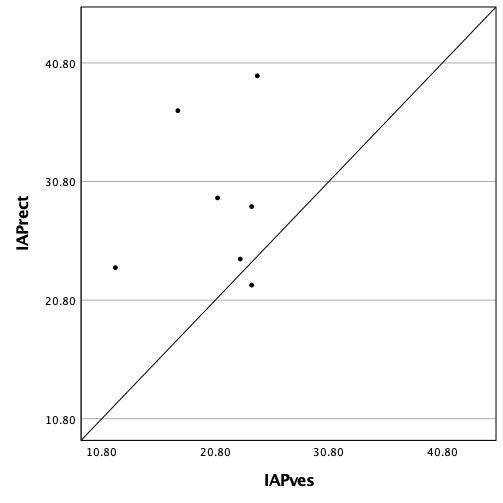


**ESM Figure 3.** Bland and Altman plots for IAP_ves_ and IAP_rect_ comparisons in different positions (supine, HOB45°) and with/without abdominal Pressure belt (n=42).

Panel A. Bland and Altman plot for IAP_ves_ and IAP_rect_ comparisons in supine position (n=14)

Panel B. Bland and Altman plot for IAP_ves_ and IAP_rect_ comparisons in HOB 45° position (n=13)

Panel C. Bland and Altman plot for IAP_ves_ and IAP_rect_ comparisons in supine position with abdominal pressure belt (n=8)

Panel D. Bland and Altman plot for IAP_ves_ and IAP_rect_ comparisons in HOB 45° position with abdominal pressure belt (n=7)
